# Prenatal Diagnosis of True Fetal Mosaicism with Small Supernumerary Marker Chromosome Derived from Chromosome 16 by Funipuncture and Molecular Cytogenetics Including Chromosome Microarray

**DOI:** 10.3390/diagnostics11081457

**Published:** 2021-08-12

**Authors:** Tien-Yu Yao, Wan-Ju Wu, Kim-Seng Law, Mei-Hui Lee, Shun-Ping Chang, Dong-Jay Lee, Wen-Hsiang Lin, Ming Chen, Gwo-Chin Ma

**Affiliations:** 1 Department of Obstetrics and Gynecology, Changhua Christian Hospital, Changhua 50006, Taiwan; 182988@cch.org.tw (T.-Y.Y.); crystalwu835@gmail.com (W.-J.W.); 2 PhD Programs in Translational Medicine, National Chung Hsing University, Taichung 40227, Taiwan; 3 Department of Genomic Medicine and Center for Medical Genetics, Changhua Christian Hospital, Changhua 50046, Taiwan; 29561@cch.org.tw (M.-H.L.); 70914@cch.org.tw (S.-P.C.); 4 Department of Obstetrics and Gynecology, Tung’s Taichung MetroHarbor Hospital, Taichung 43344, Taiwan; kimsenglaw@gmail.com; 5 Department of Nursing, Jenteh Junior College of Medicine, Nursing and Management, Miaoli 35665, Taiwan; 6 Department of Life Science, National Chung Hsin University, Taichung 40227, Taiwan; 7 Research Department, Changhua Christian Hospital, Changhua 50006, Taiwan; 118862@cch.org.tw; 8 Welgene Biotechnology Company, Nangang Business Park, Taipei 11503, Taiwan; 397620cch@gmail.com; 9 Department of Obstetrics and Gynecology, College of Medicine, National Taiwan University, Taipei 10041, Taiwan; 10 Department of Medical Genetics, National Taiwan University Hospital, Taipei 10041, Taiwan; 11 Department of Molecular Biotechnology, Da-Yeh University, Changhua 51591, Taiwan; 12 Department of Medical Science, National Tsing Hua University, Hsinchu 30013, Taiwan

**Keywords:** sSMC(16), mosaicism, chromosome microarray analysis, chromosome 16p11.2 duplication syndrome, prenatal counseling, prenatal diagnosis

## Abstract

This study examined the molecular characterization of a prenatal case with true fetal mosaicism of small supernumerary marker chromosome 16 (sSMC(16)). A 41-year-old female underwent amniocentesis at 19 weeks of gestation due to advanced maternal age. Chromosomal analysis for cultured amniocytes revealed a karyotype of 47,XY,+mar[4]/46,XY[16]. Spectral karyotyping and metaphase fluorescence in situ hybridization (FISH) demonstrated that the sSMC was derived from chromosome 16 (47,XY,+mar.ish der(16)(D16Z1+)[13/20]). Confined placental mosaicism was initially suspected because the prenatal ultrasound revealed a normal structure and the pregnancy was uneventful. However, interphase FISH of cord blood performed at 28 weeks of gestation showed 20% mosaicism of trisomy chromosome 16 (nuc ish(D16Z2×3)[40/200]). Chromosome microarray analysis further demonstrated 55% mosaicism of an 8.02 Mb segmental duplication at the subcentromeric region of 16p12.1p11.1 (arr[GRCh37] 16p12.1p11.1(27021975_35045499)×3[0.55]). The results demonstrated a true fetal mosaicism of sSMC(16) involving chromosome16p12.1p11.1 that is associated with chromosome 16p11.2 duplication syndrome (OMIM #614671). After non-directive genetic counseling, the couple opted for late termination of pregnancy. This case illustrated the use of multiple molecular cytogenetic tools to elucidate the origin and structure of sSMC, which is crucial for prenatal counseling, decision making, and clinical management.

## 1. Introduction

Small supernumerary marker chromosome (sSMC), which is defined as additional centric chromosome fragments too small to be identified or characterized by banding cytogenetics alone [1], occurs in 0.072–0.075% of prenatal cases and 0.044% of newborn cases [2]. sSMCs in most cases are single, but cases with multiple sSMCs can occur. A case with 3~7 sSMCs born with mild congenital anomalies was previously reported [3]; origins of the sSMCs were clarified by fluorescence in situ hybridization one by one with probe staining for chromosome-specific centromeric regions [3]. It seems that some chromosomes are more likely to be the origin of marker chromosomes when multiple sSMCs are found; for example, chromosome 6 was reported to be found in 33% of the patients having multiple sSMCs with the unknown mechanism [3]. Whether the mosaic marker chromosome is due to confined placental mosaicism or true fetal mosaicism is also a vital issue when offering nondirective but informative genetic counseling at the prenatal stage, of which serial invasive diagnosis, including repeated amniocentesis or cordocentesis (funipuncture), can be of help [4]. The introduction of chromosome microarray analysis (CMA) in the past decade also helps precisely to delineate the duplicated regions of the extra copies of genome segments constituting the marker chromosome in addition to molecular cytogenetic techniques [5,6,7,8]. A low-level mosaic trisomy 16 fetus with a favorable outcome was recently reported [9]. In this study, a combination of molecular cytogenetic tools was used for prenatal diagnosis of a case with mosaic sSMC, and the results showed the sSMC was originated from 16 (sSMC(16)) involving 16p12.1q11.1 duplication that is associated with chromosome 16p11.2 duplication syndrome (OMIM #614671) and anticipated with an unfavorable outcome.

## 2. Materials and Methods

### 2.1. Clinical Description

A 41-year-old female, gravida 2, para 1, received amniocentesis at 19 weeks of gestation due to advanced maternal age. Amniocentesis revealed a karyotype of 47,XY,+mar [4]/46,XY[16]. Spectral karyotyping (SKY) and metaphase fluorescence in situ hybridization (FISH) revealed the sSMC was derived from chromosome 16 (sSMC(16)). Karyotypes of both parents were normal, indicating a *de novo* origin of the sSMC(16). The prenatal ultrasound showed unremarkable results, including fetal anatomy, biometry, and target neurosonography. A diagnosis of fetal mosaicism was questionable because of the normal ultrasound findings. The pregnant woman initially denied follow-up genetic analyses. Until 28 weeks of gestation, she opted for cordocentesis because of the increased feeling of anxiety. Interphase FISH of cord blood confirmed a true fetal mosaicism of trisomy chromosome 16 (nuc ish(D16Z2×3)[40/200]). Chromosome microarray analysis revealed a mosaic duplication in chromosome 16p12.1p11.1, comprising the breakpoint 1–5 (BP1-BP5) of chromosome 16p11.2. The chromosomal 16p11.2 BP1-BP5 duplication is associated with chromosome 16p11.2 duplication syndrome with features of autism spectrum disorders (ASD) and other neurodevelopmental disorders [10]. After nondirective counseling, the pregnant women opted for late termination of pregnancy via hysterotomy due to previous cesarean section. The gross appearance of the abortus seemed normal. An autopsy was declined by the couple.

### 2.2. Cytogenetic and Molecular Cytogenetic Analysis of Amniocytes

Conventional cytogenetic G-banding analysis with Wright’s dye staining at the 550 bands of resolution was performed on cultured amniocytes according to the standard cytogenetic protocol. SKY using 24-color SKY probes (Applied Spectral Imaging, Carlsbad, CA, USA) and metaphase FISH using Satellite Enumeration probes (Cytocell Inc., Adderbury, Oxfordshire, UK) were further carried out to determine the origin of the sSMC detected in the cytogenetic analysis of cultured amniocytes.

### 2.3. Molecular Cytogenetic and Chromosome Microarray Analysis of Cord Blood

Interphase FISH analysis of uncultured specimen of cord blood was performed to confirm the existence and proportion of sSMC using chromosome 16 Satellite Enumeration probe encompassing the locus D16Z2 (16p11.1-q11.1) (Cytocell, Adderbury, Oxfordshire, UK), based on the molecular cytogenetic findings in cultured amniocytes. Chromosome microarray analysis (CMA) using an Agilent customer design oligonucleotide 8 × 60 K CytoScan^®^ gene chip (ID 040427) was further used to determine the entity of sSMC following the standard protocol (Chen et al., 2014). Scanned CMA images were analyzed by Feature Extraction 9.5.3 software (Agilent Technologies, Santa Clara, CA, USA), and the extracted data were processed using the Agilent Genomic Workbench 7.0 program (Agilent Technologies, Santa Clara, CA, USA). The CMA findings were described based on the reference genome version of GRCh37, following the latest guideline of An International System for Human Cytogenomic Nomenclature (ISCN2020). The clinical significance of copy number variations/sSMCs was according to the Genomic Variation Database (http://dgv.tcag.ca/dgv/app/home) (accessed on 12 July 2021), DECIPHER (https://www.deciphergenomics.org/) (accessed on 12 July 2021), and ChromosOmics-Database (http://cs-tl.de/DB/CA/sSMC/0-Start.html) (accessed on 12 July 2021).

## 3. Results

**Figure 1:**
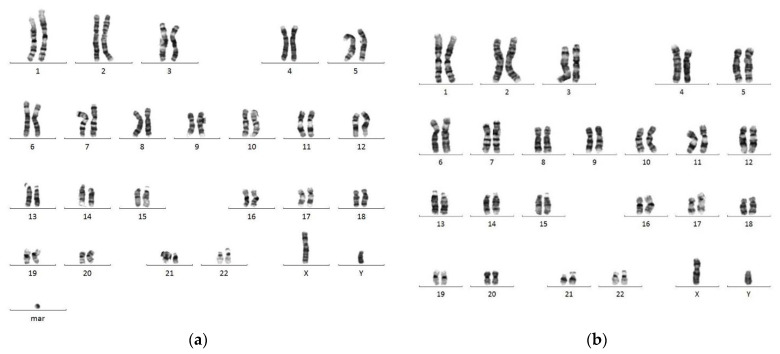
Conventional G-banding analysis of cultured amniocytes at 19 weeks of gestation revealed mosaicism of a supernumerary marker chromosome (sSMC) (47,XY,+mar.[4]/46,XY[16]): (**a**) abnormal male karyotype of 47,XY,+mar; (**b**) normal male karyotype of 46,XY. mar, sSMC.

**Figure 2:**
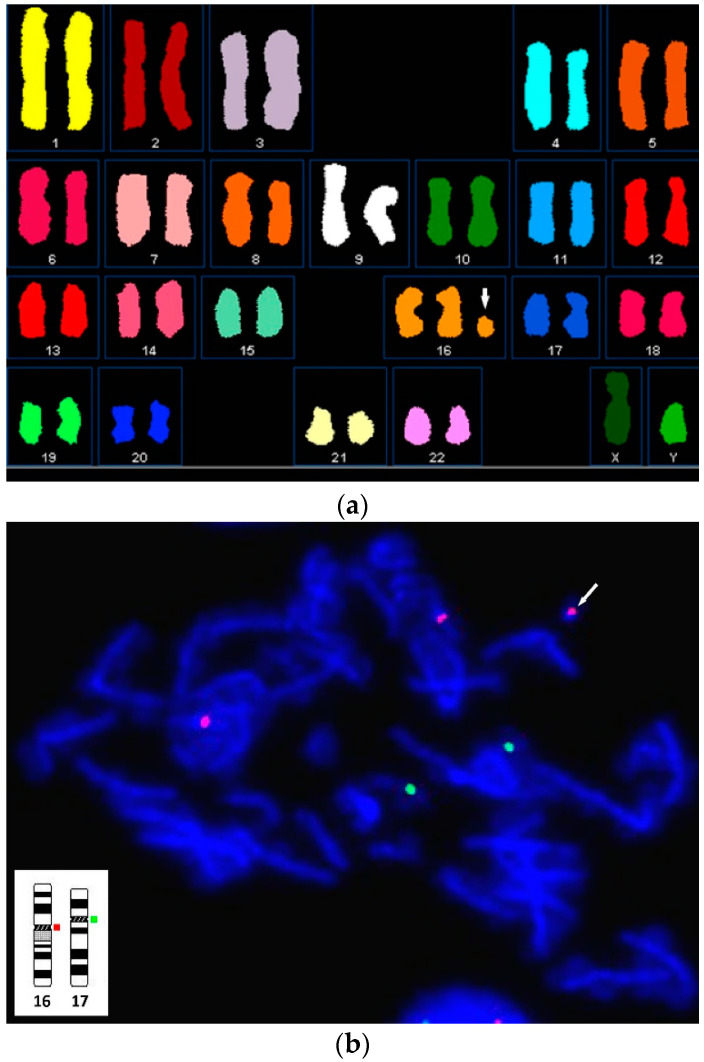
Molecular cytogenetic analyses of cultured amniocytes at 19 weeks of gestation: (**a**) spectral karyotyping (SKY) using 24-color SKY probes (Applied Spectral Imaging, Carlsbad, CA, USA) showed that the sSMC is derived from the chromosome 16 (sSMC(16)) (47,XY,+mar.ish der(16)(D16Z1+)); (**b**) metaphase fluorescence in situ hybridization (FISH) using Satellite Enumeration probes (Cytocell Inc. Adderbury, Oxfordshire, UK) confirmed the sSMC is of sSMC(16). Probes specific for the centromeres of chromosome 16 (D16Z2, corresponding to 16p11.1-q11.1) and 17 (D17Z1, corresponding to 17p11.1-q11.1) are labeled with red and green, respectively.

**Figure 3:**
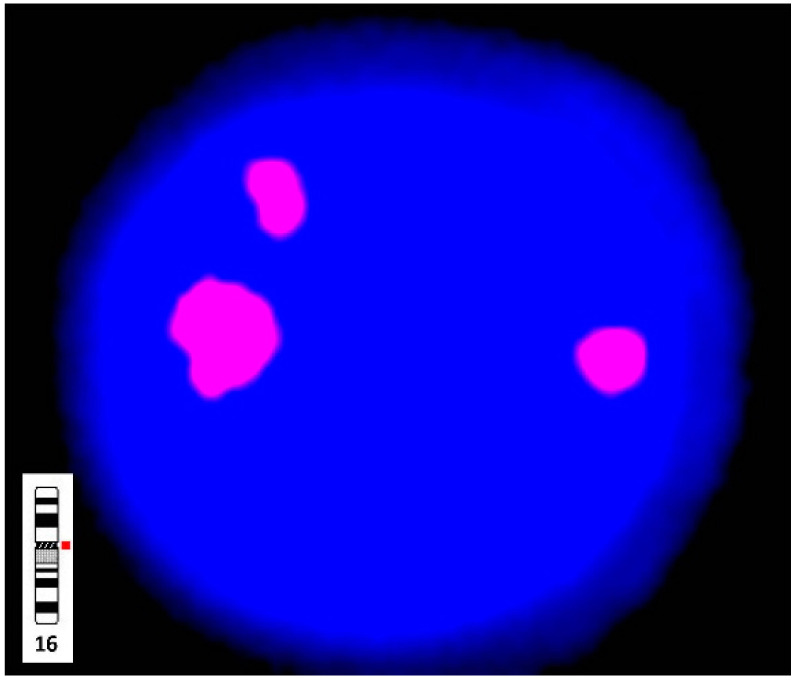
Interphase FISH analysis of cord blood performed at 28 weeks of gestation using Satellite Enumeration probes (Cytocell Inc. Adderbury, Oxfordshire, UK) confirmed a true fetal mosaicism of trisomy chromosome 16 (nuc ish(D16Z2×3)[40/200]). The probe specific for the centromere of chromosome 16 (D16Z2, corresponding to 16p11.1-q11.1) is labeled with red.

**Figure 4:**
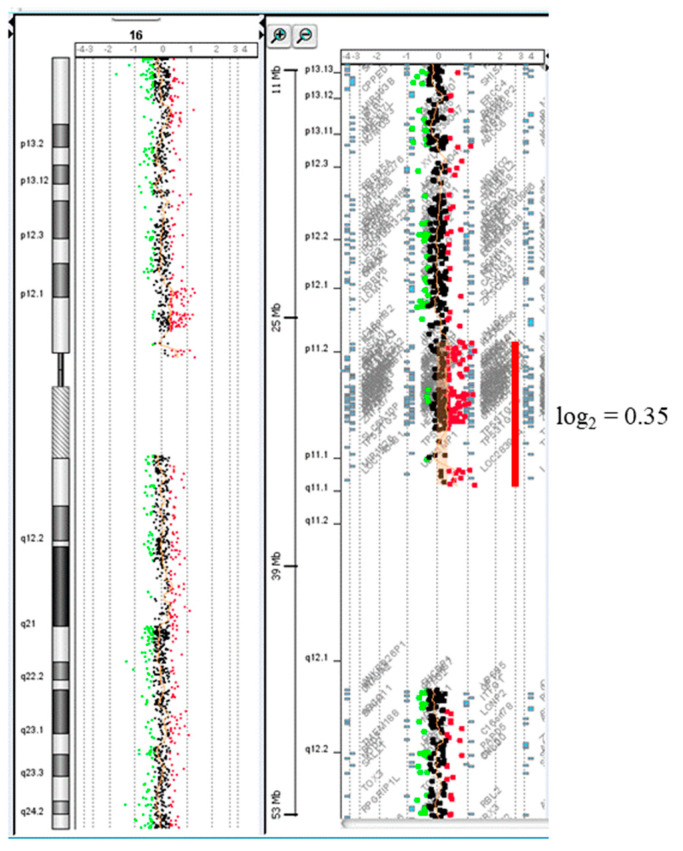
Chromosome microarray analysis using CytoScan gene chip (Agilent customer design ID 040427, Changhua Christian Hospital, Changhua, Taiwan) on blood cord demonstrated 55% mosaicism (inferred from the mean log_2_ ratio of 0.35) of 16p12.1p11.1 duplication with 8.02 Mb in size (arr[GRCh37]16p12.1p11.1(27021975_35045499)×3[0.55]).

Chromosomal karyotypic analysis of cultured amniocytes carried out at 19 weeks of gestation showed a mosaic karyotype of 47,XY,+mar [4]/46,XY[16] (Figure 1). SKY and metaphase FISH revealed 65% of cultured amniocytes had an extra sSMC(16) with a karyotype of 47,XY,+mar.ish der(16)(D16Z1+)[13/20] (Figure 2). Interphase FISH of cord blood performed at 28 weeks of gestation showed 20% mosaicism of trisomy chromosome 16 (nuc ish(D16Z2×3)[40/200]) (Figure 3). CMA of cord blood revealed a result of arr[GRCh37] 16p12.1p11.1(27021975_35045499)×3 (mean log_2_ ratio = 0.35), indicating a 8.02 Mb segmental duplication at the subcentromeric region of 16p12.1p11.1 encompassing 139 genes and covering the BP1-BP5 region of chromosome 16p11.2 (delineating by the genomic position of 28.4–30.2 Mb; GRCh37) with 55% mosaicism for genomic imbalance (Figure 4). Chromosomal analysis of cord blood also showed 20% of mosaicism of sSMC(16) (47,XY,+mar[4]/46,XY[16]).

## 4. Discussion

Chromosome 16 is featured as one of the highest degrees of segmental duplication among human autosomes. Nearly 9% of genome-wide human duplication sequences map to this chromosome [11]. Segmental duplications are hotbeds for genomic rearrangements [12], a possible mechanism related to sSMC formation [1]. So far, 100 cases of sSMC(16) with variable chromosome 16 rearrangements were recorded in the ChromosOmics-Database. The case presented here had a copy number gain of 8.02 Mb in size, covering the chromosome 16p12.1p11.1 BP1-BP5 region. Actually, the size of the sSMC(16) was expected to be larger than that estimated in CMA because array probes for centromeres/heterochromatins are avoided to be used due to their repetitive nature and the result of metaphase FISH showed the sSMC(16) covered the centromeric region of chromosome 16 (Figure 2b). The chromosome 16p11.2 has been regarded as a locus susceptible to recurrent disease-associated rearrangements mediated by a complex set of segmental duplications that recently arose during human evolution [13]. In ChromosOmics-Database, the chromosome 16p12 and part of the 16p11.2 region is considered as critical regions with clinical sign of sSMC(16). To the best of our knowledge, no similar sSMC(16) case was previously reported in the literature and public record databases.

Trisomy 16 (T16) is the most frequent autosomal anomaly seen in early spontaneous abortions, accounting for 12.7–15% of first-trimester pregnancy losses [14,15]. Full T16 is considered to be lethal, but fetuses with mosaic or partial T16 may survive with various outcomes, ranging from nearly normal to severe handicapped [14]. Recent studies showed that partial trisomies with contiguous gene duplications of BP1-BP5 and the proximal BP4-BP5 region of chromosome 16p (genomic position of 28.4–30.2 Mb and 29.6–30.2 Mb, respectively; GRCh37) are responsible for chromosome 16p11.2 duplication syndrome, which manifests a wide variety of neuropsychiatric phenotypes, growth anomalies, skeletal anomalies, and less-frequent congenital anomalies [16]. It was reported that recurrent duplications and deletions of approximately 555 Kb at 16p11.2 confer susceptibility to ASD in up to 1% of ASD patients [17]. This observation is in concordance with what is observed in individuals with 16p11.2 duplications and deletions who frequently suffer from neurodevelopmental disorders (NDDs) [18]. Particularly, cases with 16p11.2 duplication are more likely to have neurodevelopmental problems (especially attention deficit hyperactivity disorder), epilepsy, and specific speech disorders [18]. In addition to NDD, 16p11.2 duplication is also related to psychiatric disorders; for example, patients with 16p11.2 duplication have a 14.5-fold increased risk of schizophrenia [19]. Notably, copy number variations (CNVs) at 16p11.2 cause mirror phenotypes, in which deletion carriers present obesity and macrocephaly, while the duplication group has a tendency for being underweight and statistically significant microcephaly [16,18]. The distal 16p11.2 BP2-BP3 region (221 Kb spanning genomic position of 28.8–29.0 Mb; GRCh37) is another recurrent CNVs region. Phenotypes associated with 16p11.2 BP2-BP3 duplication are the tendency toward BMI and head circumference reduction [20].

Several genes in the BP4-BP5 region of chromosome 16p may play roles in NDD, including *QPRT* (OMIM#606248), *PRRT2* (OMIM#614386), *KCTD13* (OMIM#608947), and *DOC2A* (OMIM#604567). Levels of QPRT, involved in the catabolism of quinolinic acid, in the human brain have been postulated to be involved in the pathogenesis of neurodegenerative disorders. It was suggested that QPRT may play an important role in the pathogenesis of ASD in 16p11.2 deletion carriers [21]. *PRRT2* encoded a protein involved in synaptic transmission in central nerve system and associated with autosomal dominant familial infantile convulsions with paroxysmal choreoathetosis (OMIM # 602066), benign familial infantile seizures type 2 (OMIM # 605751), and episodic kinesigenic dyskinesia type 1 (OMIM: 128200). *KCTD13* has been suggested to drive the mirror microcephaly/macrocephaly phenotypes in a zebrafish study [22]. Additionally, deletion of *KCTD13* in mice showed reduced synaptic transmission, supporting a role of KCTD13 in the regulation of neuronal function relevant to neuropsychiatric disorders [23]. *DOC2A* encodes a calcium sensor which most probably regulates the fusion of vesicles with membranes. DOC2A can regulate spontaneous synaptic transmission and has been implicated in Ca^2+^-dependent neurotransmitter release. The abnormal protein expression of DOC2A in epileptic brain tissue may play an important role in epilepsy [24].

## 5. Conclusions

This report presented the prenatal diagnosis of a true fetal mosaicism of sSMC(16) involving chromosome 16q12.1q11.1, related to chromosome 16p11.2 duplication syndrome. By using different molecular cytogenetic tools, including SKY, FISH, and CMA, the origin and structure of the abnormal chromosome were determined that provides crucial information for prenatal counseling and clinical management.

## Data Availability

Data are available upon request.
